# Expansion of the genetic and phenotypic spectrum of hereditary spastic paraplegia caused by *ABHD16A* gene variants: an integrated analysis based on novel variants and literature review

**DOI:** 10.3389/fped.2025.1724515

**Published:** 2026-01-05

**Authors:** Manling He, Qiang Zhang, Shaoke Chen, Chuan Li, Bobo Xie, Qingxiu Zhao, Yiyun Huang, Xin Fan

**Affiliations:** 1 Department of Pediatric, The Second Affiliated Hospital of Guangxi Medical University, Guangxi, China; 2 Department of Genetic and Metabolic Central Laboratory, Maternal and Child Health Hospital of Guangxi Zhuang Autonomous Region, Nanning, China; 3 Department of Medical Genetics and Genomics Central Laboratory, The Second Affiliated Hospital of Guangxi Medical University, Guangxi, China

**Keywords:** *ABHD16A*, *BAT5*, developmental delay, hereditary spastic paraplegia, long-chain fattyacids

## Abstract

**Background:**

Hereditary spastic paraplegia (HSP) is a clinically and genetically heterogeneous neurodegenerative disorder. Biallelic pathogenic variants in *ABHD16A* have recently been linked to a neurodevelopmental phenotype featuring early-onset spasticity and global developmental delay.

**Objective:**

To further define the clinical and genetic spectrum of *ABHD16A*-associated disease through the characterization of a novel pediatric case and an updated literature review.

**Methods:**

We evaluated a child presenting with global developmental delay and progressive spastic paraplegia. Whole-exome sequencing (WES) was performed, and candidate variants were validated by Sanger sequencing. Clinical features were documented prospectively, and a systematic review of published cases was conducted to assess phenotypic patterns and genotype–phenotype relationships.

**Results:**

Consistent with prior reports, the core features of *ABHD16A*-related disease include global developmental delay, intellectual disability, and spastic paraplegia, often with early onset. In our patient, tandem mass spectrometry revealed elevated long-chain acylcarnitines (C16, C18:1, C18:2)—a metabolic abnormality not previously described in this condition. WES identified two novel compound heterozygous frameshift variants in *ABHD16A*: c.119delA (p.His40Leufs49) and c.559_562del (p.Pro187Cysfs29), both confirmed by Sanger sequencing and classified as pathogenic (ACMG criteria: PVS1, PM2, PM3, PP1). Our literature review identified 17 additional individuals from 9 families, enabling a refined clinical delineation: most patients exhibited motor and speech delay, axial hypotonia evolving into spasticity, and variable degrees of cognitive impairment.

**Conclusions:**

To our knowledge, this is the first reported case of *ABHD16A*-related neurodevelopmental disorder in a Chinese patient. We provide a detailed phenotypic characterization and an updated review of the published literature to support clinical recognition and genetic diagnosis of this emerging condition.

## Introduction

The *ABHD16A* gene-formerly designated BAT5(Human lymphocyte antigen B-associated transcript 5) (1), encodes a protein that contains an *α*/*β* hydrolase domain. It spans 21 exons on chromosome 6p21.33 (2). *ABHD16A* is expressed in various cell types, including skeletal muscle, brain, cardiac muscle, and testis, etc (3, 4). *ABHD16A* plays a critical role in lipid metabolism and signaling. It functions primarily as a phosphatidylserine (PS) lipase, catalyzing the hydrolysis of PS to generate lysophosphatidylserine (lyso-PS). Additionally, it exhibits monoacylglycerol lipase activity towards certain medium- and long-chain fatty acid substrates (3).

Biallelic pathogenic variants in *ABHD16A* have been associated with a neurodevelopmental disorder characterized by intellectual disability and hereditary spastic paraplegia (7–9). Additional studies suggest roles in immune regulation (5, 6, 10) and Kawasaki disease (11).

Here, we describe the first pediatric case of *ABHD16A*-related disorder reported in mainland China. By integrating detailed clinical and molecular findings with an updated synthesis of the published literature (17 patients from 9 families), we aim to improve early recognition and diagnostic accuracy of this rare condition in clinical practice.

## Materials and methods

### Clinical date, tandem mass spectrometry and genetic analysis

We inquired in detail and recorded the patient's medical history. Meanwhile, we have essentially completed the relevant examinations for the patient and conducted follow-up visits to her. In addition, we also performed genetic testing and analysis on the patients through WES and Sanger sequencing.

Tandem Mass Spectrometry: Fasting peripheral blood samples were collected from the patient onto filter paper to generate dried blood spots (DBS), which were subsequently analyzed by tandem mass spectrometry. Targeted quantitative analysis of amino acids and acylcarnitines was performed using an in-house, clinically validated Laboratory Developed Test (LDT) on a Shimadzu LCMS-8040 triple quadrupole liquid chromatography–tandem mass spectrometer (Shimadzu Corporation, Kyoto, Japan). This LDT was developed, validated, and implemented for clinical diagnostic use in Minlusi (Beijing) Medical Laboratory. The assay participates in external quality assessment schemes (e.g., ERNDIM, US CDC).

Whole-exome sequencing (WES): Genomic DNA was extracted from peripheral blood samples of the proband and her parents. Whole-exome sequencing was performed on the Illumina NovaSeq 6,000 platform using the IDT xGen Exome Research Panel v2.0 for target enrichment and library construction, with paired-end sequencing yielding >98% of the target region covered at ≥20× dept.

In the subsequent step, the process proceeds to bioinformatic analysis pipeline.

Variant Calling and Filtering—Raw sequencing reads were processed using the Genome Analysis Toolkit (GATK). Specifically, alignment to the UCSC human reference genome (hg19/GRCh37) and variant calling were performed with GATK HaplotypeCaller. Variants were filtered against population databases—including gnomAD, the NHLBI Exome Sequencing Project (ESP), and the 1,000 Genomes Project (1000G)—as well as an in-house database of ethnically matched healthy controls; variants with allele frequencies >3% in any of these cohorts were excluded. Non-functional variants (e.g., synonymous substitutions and non-coding variants outside canonical splice sites) were removed. Remaining candidate variants were prioritized based on in silico pathogenicity predictions (SIFT, PolyPhen-2, and M-CAP), clinical phenotype correlation, and segregation analysis within the family. Final classification adhered to the ACMG/AMP 2015 guidelines for the interpretation of sequence variants, incorporating evidence from disease-specific databases (e.g., ClinVar, OMIM, HGMD) and peer-reviewed literature. Copy number variants (CNVs) were assessed using a complementary approach: normalized exome sequencing depth was first calculated per sample using GATK tools; subsequently, CNV calling was performed with XHMM, comparing the proband's trio data against an internal cohort of control exomes to identify rare, family-specific CNVs.

Variant Annotation and Prioritization—Functional annotation of variants was performed using three independent tools: ANNOVAR, Oncotator, and Ensembl Variant Effect Predictor (VEP). Candidate pathogenic variants were prioritized by cross-referencing curated databases of known or putative disease-associated variants, including ClinVar, OMIM, and HGMD. To enrich for rare, potentially deleterious alleles, variants were further filtered against large-scale population frequency resources—primarily gnomAD and the Exome Aggregation Consortium (ExAC)—retaining only those with minor allele frequencies (MAF) < 1% in global and population-matched subsets.

Allele frequency filtering was performed using the Genome Aggregation Database (gnomAD), which comprises aggregated exome sequencing data from 125,748 unrelated individuals across diverse global populations. For variants absent or extremely rare in gnomAD, we additionally consulted the in-house database of 1,000 ethnically matched (Chinese) healthy controls, generated from whole-exome sequencing in our center. Allele frequencies were evaluated in both the global gnomAD cohort and the population-matched subgroups (EAS and in-house controls); variants with minor allele frequency (MAF) ≥ 1% in any of these datasets were excluded from downstream analysis.

Sanger sequencing: The sequencing primer for the *ABHD16A* c.119delA(p.His40Leufs*49) variant is 5'-CACCCTCACTCTGAACCTAA-3' and 5'-TACTCCTAATCTCACCCCTC-3'. The sequencing primer of the *ABHD16A* c.559_562del (p.Pro187Cysfs*29) variant is 5'-GACCTCTCTGAGCCTCTTTT-3' and 5'-CCTATCCTTCCCTGCATCTT-3'.

### Literature review

We conducted a literature search using the keywords “*ABHD16A*,” “BAT5,” “spastic paraplegia,” “developmental delay,” and “language regression,” across the CNKI, Wan fang, VIP, and PubMed databases for publications up to December 2024. Subsequently, we performed an analysis on the retrieved content.

## Result

### Clinical information

The proband is a 13-month-old girl born to non-consanguineous parents. She was referred to our pediatric genetics and endocrinology clinic at 12 months of age for global developmental delay, noted since early infancy. The patient was able to raise her head at the age of 6 months. Assessments using the Gesell Developmental Schedules (GDS) at both 6 months and 12 months, indicated delayed intellectual development. At 6 months old, her developmental milestones were as follows: social maturity (SM) was borderline, gross motor function was equivalent to 3.9 months, fine motor function equivalent to 3.6 months, adaptive behavior equivalent to 3.7 months, language skills equivalent to 3.3 months, and social skills equivalent to 3.3 months. By 12 months, the GDS results showed SM was still borderline, gross motor function equivalent to 5.9 months, fine motor function to 7 months, adaptive behavior to 6.5 months, language skills to 7.9 months, and social skills to 6.1 months. She began rehabilitation at 6 months age. By the age of 1 year, she could roll over, sit with support, and vocalize in response to stimuli. Her current dietary intake is adequate; however, she has trouble in falling asleep.

Birth and Personal History: The patient was born full-term via vaginal delivery with a birth weight of 2.8 kg and a length of 48 cm. There was no history of asphyxia or neonatal jaundice. Feeding difficulties were noted during the neonatal period, but there were no incidents of choking or aspiration.

Physical Examination: At 1 year and 1 month of age, the patient was alert and responsive. Anthropometric measurements revealed significant growth impairment: height 72.5 cm (−1.0 SD), weight 6.78 kg (−3.2 SD), and head circumference 45.7 cm (+0.5 SD), all referenced to standardized growth charts for Chinese children and adolescents (12, 13). The anterior fontanelle measured 3 × 3 cm. Notable physical features included a prominent forehead, low-set ears, high palate, and micrognathia. Cardiopulmonary and abdominal examinations were unremarkable. Hypotonia was observed in all four limbs.

**Figure 1:**
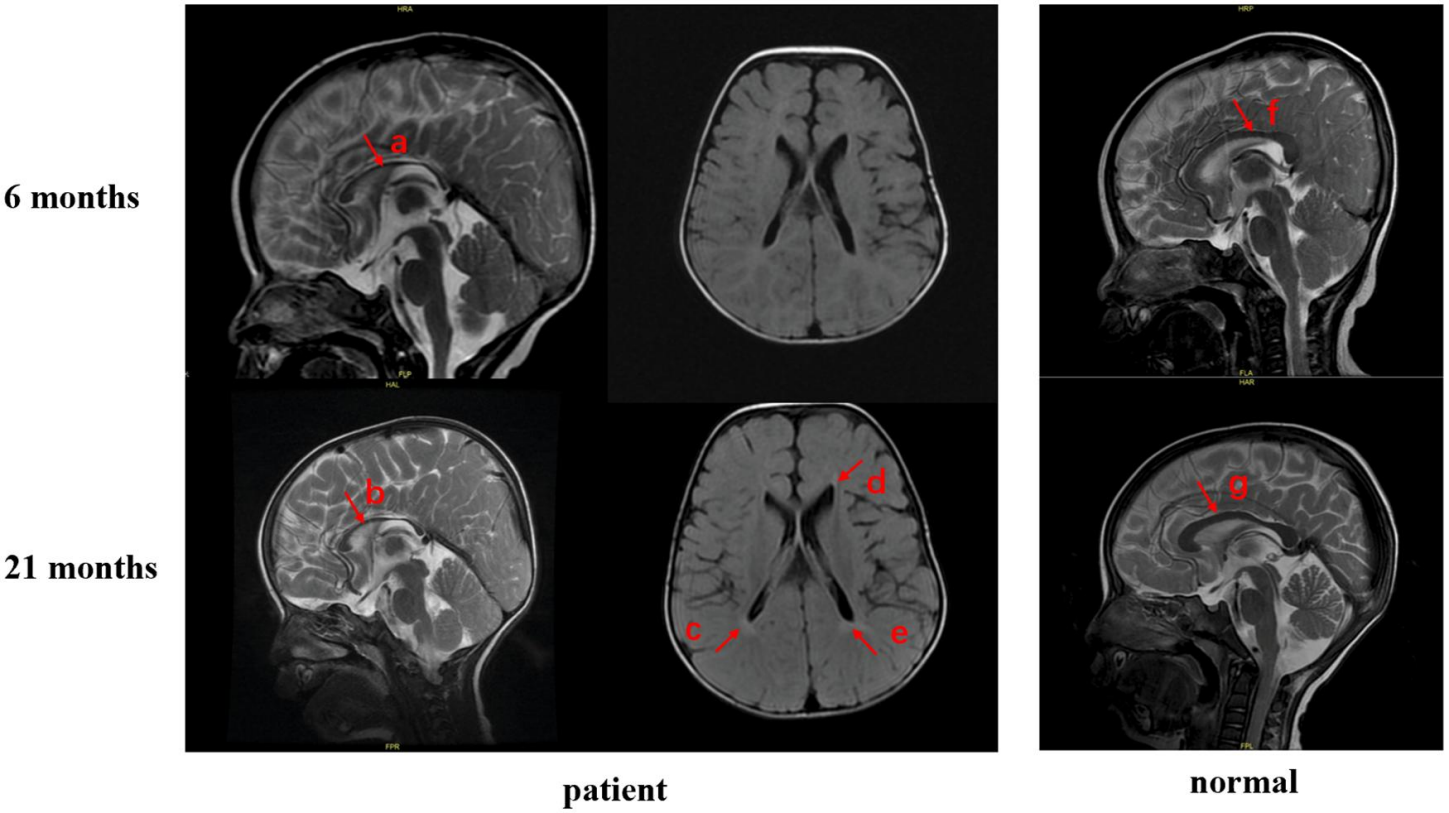
Brain MRI. **(a)** Corpus callosum of the patient at 6 months of age. **(b)** Corpus callosum of the patient at 21 months of age. **(c–e)** Abnormal signal intensity in the periventricular region of the patient at 21 months of age. **(f-g)** Corpus callosum of a healthy age-matched control.

Laboratory and Imaging Findings: Liver function, renal function, lipid profile, cardiac enzymes, insulin levels, plasma ammonia, and random blood glucose levels were all within normal limits. At 6 months age, brain MRI revealed a thin corpus callosum, indicating possible hypoplasia. A follow-up MRI at 21 months showed persistence of a thin corpus callosum and abnormal signal intensity adjacent to the posterior horns of the lateral ventricles, suggesting corpus callosum hypoplasia and delayed myelination (Figure 1). At 2 year and 11 months of age, tandem mass spectrometry analysis of dried blood spots revealed mild but consistent elevations in multiple long-chain acylcarnitines: acetylcarnitine (23.92 µmol/L; ref: 0–21.60), palmitoylcarnitine (1.36 µmol/L; ref: 0–1.27), stearoylcarnitine (0.98 µmol/L; ref: 0–0.89), and hexacosanoyl-LPC (0.62 µmol/L; ref: 0.096–0.490), all exceeding their respective upper limits of normal.

### Genetic analysis

**Figure 2:**
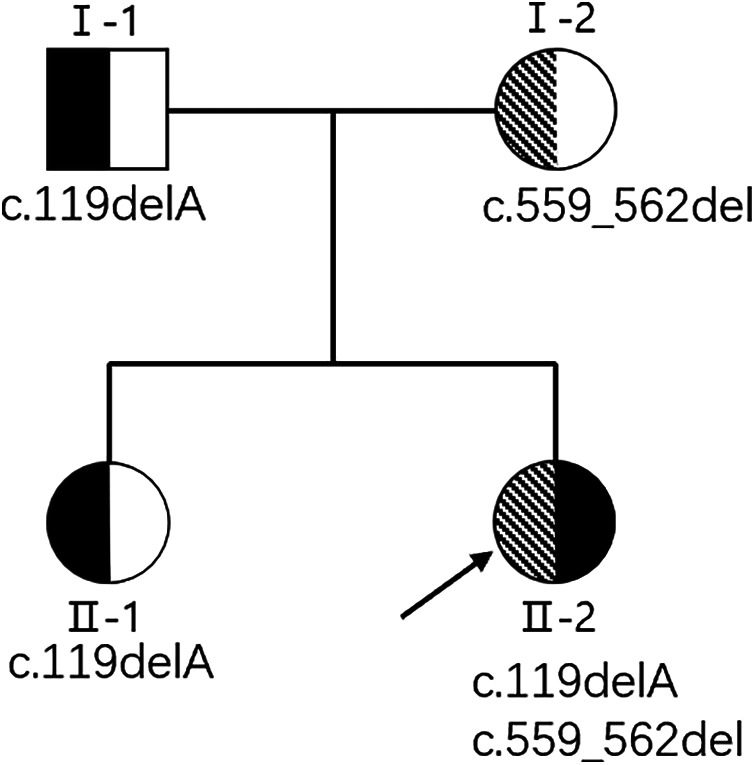
Pedigree.

**Figure 3:**
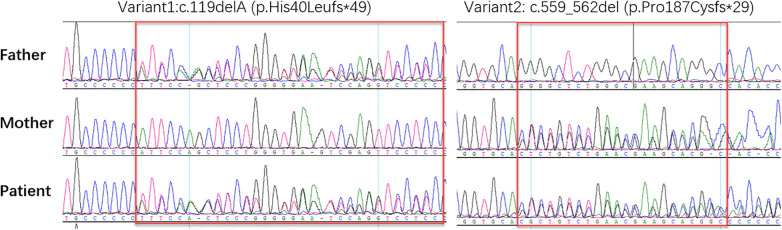
Sanger sequencing results: sanger sequencing showed that the patient carried c.119delA (p. His40Leufs * 49) from her father and c.559_562del (p. Pro187Cysfs * 29) from her mother.

We performed whole-exome sequencing on the patient, and identified compound heterozygosity for two variants: c.119delA(p.His40Leufs*49) variant and c.559_562del(p.Pro187Cysfs*29) variant in the *ABHD16A*(NM_021160.3) (Figure 2). Sanger sequencing verified the authenticity and parental origin of these mutations (Figure 3). The c.119delA (p.His40Leufs*49) variant was inherited from her father while the c.559_562del (p.Pro187Cysfs*29) variant was inherited from her mother. Neither the c.119delA (p.His40Leufs*49) nor the c.559_562del (p.Pro187Cysfs*29) variant has been documented in population databases. Both variants are predicted to induce nonsense-mediated mRNA decay, which may impair the function of the encoded protein. According to ACMG guidelines, the c.119delA (p.His40Leufs*49) variant was classified as likely pathogenic based on criteria PVS1 and PM2. Given the autosomal recessive inheritance pattern of the disease and the identification of a likely pathogenic variant in trans (c.559_562del, p.Pro187Cysfs*29), the latter variant was classified as pathogenic based on criteria PVS1, PM2, and PM3. Both mutations have not been reported.

### Follow-up

At the age of 2, she was able to articulate “dad” and “mom” and recognized family members. However, her language development has subsequently regressed since then. By the age of 3 years, lower limb spasticity emerged. Currently, at age of 4 years and 4 months, she can stand with support and produce unintentional vocalizations. Both upper and lower limb spasticity are present. A physical examination revealed hyperreflexia and positive ankle clonus.

### Literature review

**Figure 4:**
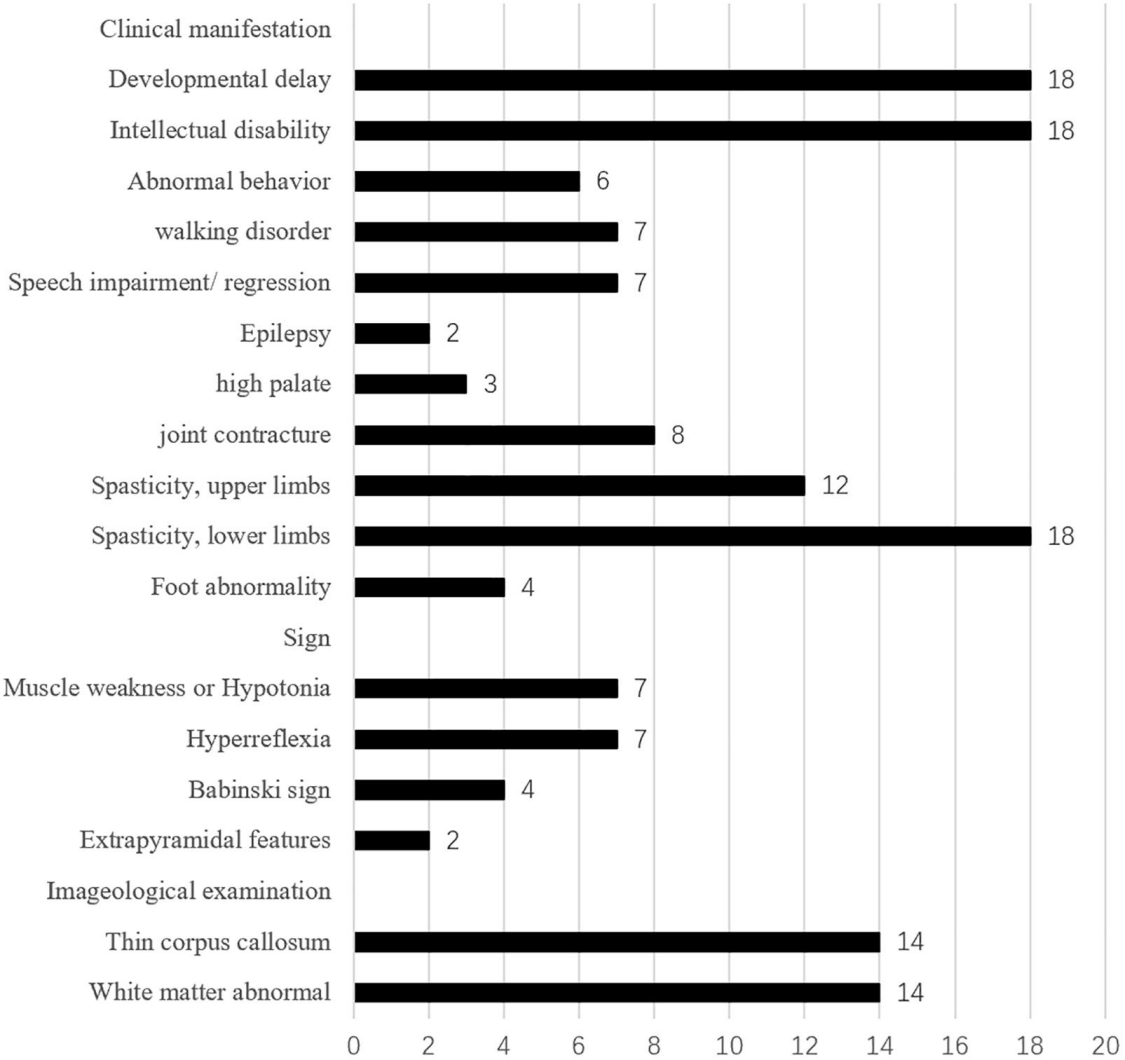
Statistical analysis of clinical manifestations in patients with *ABHD16A* gene variants.

This search yielded three articles that reporting on nine families, encompassing 17 patients with *ABHD16A* gene variants (7–9). The reported clinical symptoms predominantly included developmental delay, intellectual disability, language impairment or regression, limb spasticity, thin corpus callosum, and white matter abnormalities, with progressive worsening over time (Figure 4 and Table 1). The clinical manifestations observed in the patient in this study are align closely with these phenotypes.

**Table 1:** Clinical features of the present patient and previously reported patients with ABHD16A gene variants.

Patient ID	Yahia et al. 2021 (*n* = 4)	Lemire et al. 2021 (*n* = 11)	Noriko Miyake et al. 2022 (*n* = 2)	This study	Total (*n* = 18)
Mean age of publication	7-10 year	4-12 year	10–15 year	1.08 year	
Clinical manifestation					
First symptom	Developmental delay, lower limbs stiffness	NA	Developmental delay, spasticity	Developmental delay	
Developmental delay	4/4	11/11	2/2	+	18/18（100%）
Intellectual disability	4/4	11/11	2/2	+	18/18（100%）
Abnormal behavior	2/4	2/11	2/2	-	6/18（33%）
Walking disorder	4/4	NA	2/2	+	7/7（100%）
Speech impairment/ regression	4/4	NA	2/2	+	7/7（100%）
Epilepsy	NA	2/11	NA	-	2/11（18%）
High palate	NA	NA	2/2	+	3/3（100%）
Joint contracture	NA	8/11	NA	-	8/12（67%）
Spasticity, upper limbs	2/4	7/11	2/2	+	12/18（67%）
Spasticity, lower limbs	4/4	11/11	2/2	+	18/18（100%）
Foot abnormality	2/4	NA	2/2	-	4/7（57%）
Sign					
Muscle weakness or Hypotonia	3/4	1/1	2/2	+	7/8（88%）
Hyperreflexia	4/4	NA	2/2	+	7/7（100%）
Babinski sign	2/4	NA	2/2	-	4/7（57%）
Extrapyramidal features	2/4	NA	0/2	-	2/7（29%）
Imaging examination					
Thin corpus callosum	2/2	9/11	2/2	+	14/16（88%）
White matter abnormal	2/2	10/11	1/2	+	14/16（88%）

NA: not available, x/x: number of patients/check number.

**Figure 5:**
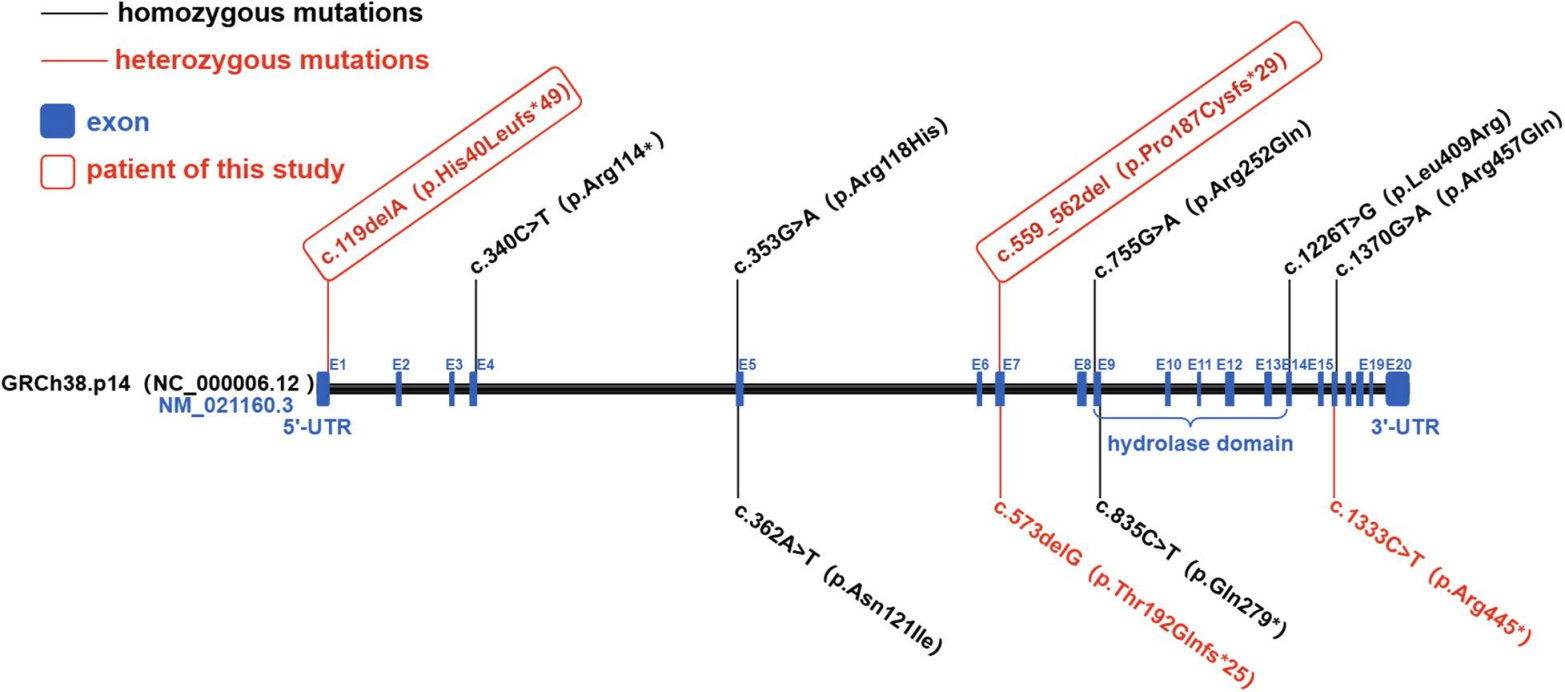
Mutation sites of the *ABHD16A* gene.

Based on the current patient and previously reported patients (7–9), a total of 11 *ABHD16A* gene variants have been identified (Figure 5). The majority of these variants are missense variants (45.45%, 5/11), nonsense variants (27.27%, 3/11), and frameshift variants (27.27%, 3/11). Among the reported patients, 16 were homozygous for pathogenic variants, while only 2 exhibited compound heterozygous variants. The patients originated from seven geographically distinct populations: Sudan, France-Canada, Armenia, Europe, Egypt, Pakistan, and Chile, with consanguineous pedigrees particularly prevalent in Sudanese, Armenian, Egyptian, and Pakistani families. Notably, whole-exome sequencing revealed no shared pathogenic variants across different pedigrees, and no hotspot or recurrent mutations. In the present patient, the identified mutations, c.119delA and c.559_562del, have not been previously reported and were not documented in public databases.

## Discussion

All 18 patients harboring biallelic pathogenic *ABHD16A* variants presented with global developmental delay (GDD) and intellectual disability (ID) (18/18, 100%). Progressive spasticity developed in every case (18/18, 100%), typically beginning in the lower limbs (mean onset age: ∼3 years) and later extending to the upper limbs, with documented progression in severity over time (7). Language regression was observed in all seven patients with available longitudinal language assessments (7/7, 100%), indicating it may be a characteristic feature of this disorder.

Additional neurological features included gait disturbance (16/18, 89%), hypotonia or generalized muscle weakness (14/18, 78%), and hyperreflexia (15/18, 83%). Brain MRI, performed in 15 patients, consistently showed structural abnormalities: a thin corpus callosum (12/15, 80%) and periventricular white matter changes—most commonly adjacent to the anterior and/or posterior horns of the lateral ventricles (13/15, 87%). Symptom onset occurred during infancy in all patients (≤12 months). While early motor and cognitive milestones were delayed, a phase of developmental stagnation or regression, particularly affecting expressive language—became evident in the majority as spasticity progressed.

*ABHD16A* gene encodes a phosphatidylserine (PS)-specific lipase that hydrolyzes PS to produce lysophosphatidylserine (lyso-PS) and a free fatty acid (10, 14). PS is a glycerophospholipid consisting of a glycerol backbone, two fatty acyl chains, and a serine headgroup. Structural diversity of PS arises from variation in acyl chain length, degree of unsaturation, and sn-positioning—yielding over 2,700 theoretically possible PS species (15). Among these, 1-stearoyl-2-docosahexaenoyl-PS (18:0, 22:6) is the most abundant species in the human brain (16). PS constitutes ∼7%–8% of total phospholipids in the healthy brain (17) and plays key roles in myelination and synaptic signaling (16, 18).

Myelin, a multilayered lipid-rich membrane produced by oligodendrocytes around axons, forms the bulk of cerebral white matter (19, 20). Disruption of myelin integrity often manifests on MRI as white matter hyperintensities (21). Notably, PS levels exhibit dynamic spatiotemporal regulation during demyelination and remyelination: total brain PS does not increase during active demyelination, though select species rise; in contrast, global PS declines during peak demyelination and remyelination phases (22). The mechanisms underlying these shifts remain unclear.

In patient-derived fibroblasts with *ABHD16A* loss-of-function variants, multiple PS species accumulate, accompanied by reduced long-chain lyso-PS (8). However, *ABHD16A* −−/−− mice show decreased brain lyso-PS without changes in total PS (10), though cerebellar enrichment of specific long-chain acyl-PS species (e.g., 18:0,22:6-PS) has been reported (23). These findings suggest that *ABHD16A* deficiency does not uniformly elevate total PS but may alter the acyl-chain composition of PS pools—particularly those containing long-chain polyunsaturated fatty acids.

Given PS's role as a major carrier of long-chain fatty acids in neural membranes, *ABHD16A*-mediated remodeling could influence local fatty acid availability. Yet, without comprehensive metabolic profiling (e.g., targeted lipid omics in CSF, plasma, and postmortem brain tissue), the functional link between *ABHD16A* dysfunction and long-chain fatty acid metabolism remains speculative and warrants further investigation.

PS is a major structural and signaling component of myelin membranes; disruption of PS homeostasis could therefore impair myelin formation or stability. A secondary effect on lipid metabolism—particularly altered handling of long-chain fatty acids—is also possible, given that abnormal fatty acid profiles (e.g., elevated very-long-chain fatty acids) disrupt oligodendrocyte maturation and myelination in peroxisomal disorders (24). To date, however, none of the 18 reported *ABHD16A*-related cases have undergone systematic metabolic workup (e.g., plasma acylcarnitine, very-long-chain fatty acid, or phospholipid profiling), making it difficult to assess whether observed biochemical shifts are disease-specific or epiphenomenal. Targeted lipidomic studies in future patients are needed to clarify this.

## Conclusion

In summary, the hallmark clinical features of *ABHD16A* gene related observed in affected individuals include global developmental delay, intellectual disability, progressive limb spasticity, and thin corpus callosum and white matter abnormalities as seen on brain MRI, metabolic disturbances in long-chain fatty acid metabolism maybe a novel phenotypic aspect of this condition.

*ABHD16A* encodes a brain-specific phosphatidylserine (PS) lipase that plays a critical role in lipid metabolism and homeostasis by catalyzing the hydrolysis of PS to generate lysophosphatidylserine (lyso-PS). Although neither phosphatidylserine (PS) nor lysophosphatidylserine (lyso-PS) has been shown to directly regulate specific neural circuits, dysregulation of the PS–lyso-PS axis could potentially contribute to alterations in myelination and demyelination — processes that are thought to play an important role in the development and maintenance of higher cognitive and communicative functions.The underlying molecular and cellular mechanisms remain to be elucidated.

## Data Availability

The datasets presented in this study can be found in online repositories. The names of the repository/repositories and accession number(s) can be found below: https://www.ncbi.nlm.nih.gov/, PRJNA1338076.
