# Supplementary figures and images for: Expansion of the genetic and phenotypic spectrum of hereditary spastic paraplegia caused by ABHD16A gene variants: an integrated analysis based on novel variants and literature review
Source: Front Pediatr. 2026 Jan 5;13:1724515. doi: 10.3389/fped.2025.1724515 (PMC12813174; doi:10.3389/fped.2025.1724515)

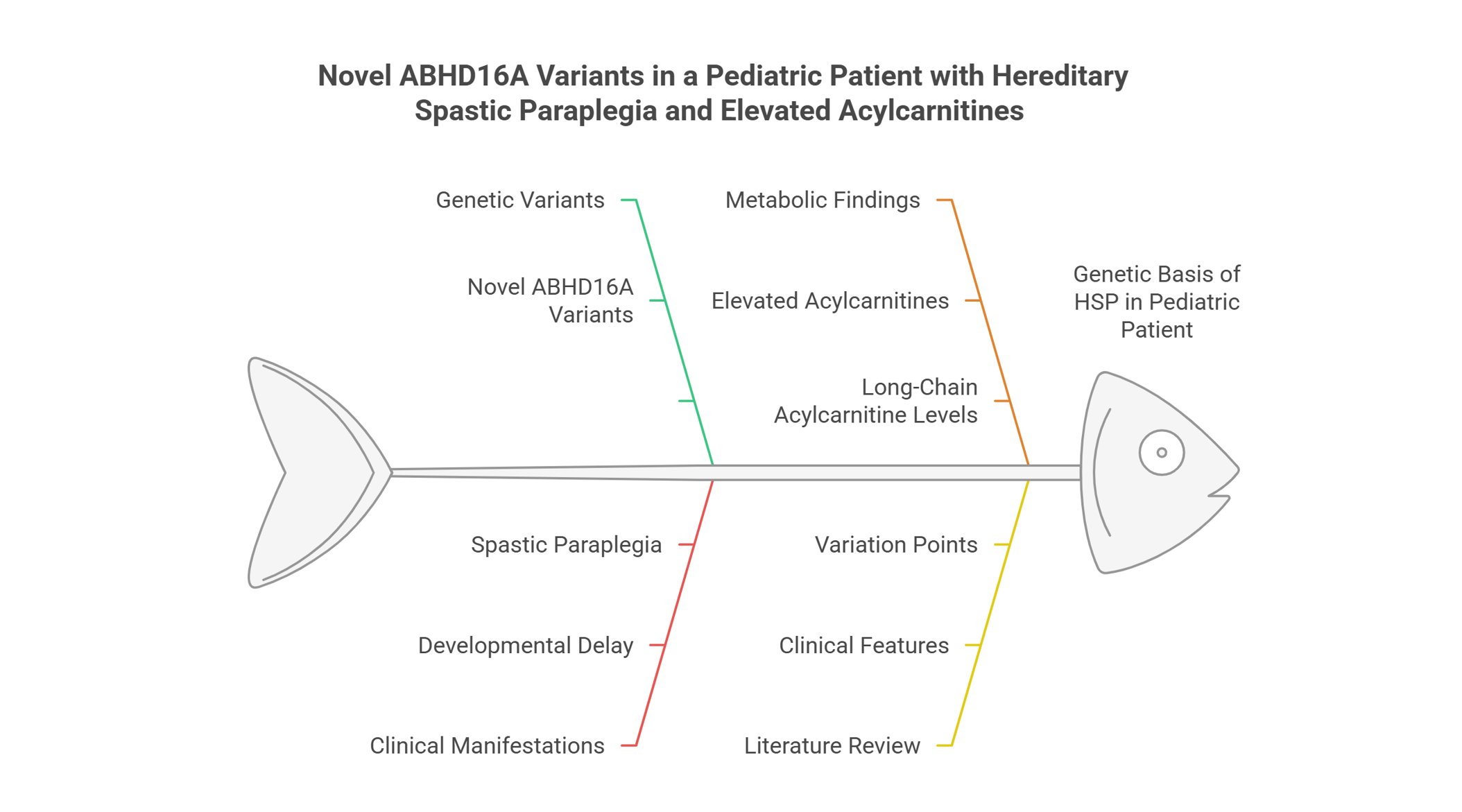

Supplement: Supplementary file 1 [file Image1.png]
